# Tobacco control in the Western Pacific region

**DOI:** 10.1016/j.lanwpc.2021.100179

**Published:** 2021-05-28

World No Tobacco Day falls on May 31, 2021. WHO has launched a year-long global campaign with the theme of Commit to Quit to mark the day. The health effects of tobacco use are devastating. According to the Global Burden of Diseases, Injuries, and Risk Factors Study 2019, published in *The Lancet,* tobacco use accounts for 8•7 million deaths, including non-smokers exposed to second-hand smoke. Mortality attributed to tobacco use has been significantly increasing over the years and is projected to rise even more. Beyond health impacts, the socioeconomic burden of tobacco is substantial and multifaceted. Tobacco use burdens societies with enormous health-care costs, losses in workforce productivity, the financial consequences of premature mortality and disability, and other indirect effects such as fires and environmental contamination.

In the otherwise exceptionally diverse Western Pacific region, the health and socioeconomic burden of tobacco use is shared by all 37 member countries. The region is home to a third of the global smoking population. In 2017, it was estimated that the tobacco-related death toll in the region exceeded 3 million people, including 460 000 non-smokers exposed to second-hand smoke. Given the over-representation of the tobacco problem in the region, it was the first of the six WHO regions to have all its member countries agree to a reduction target. In 2009, and again in 2014, all member states agreed to reduce tobacco use by 10% in the next 5 years. The selection of this target was informed by the WHO Global Action Plan for the Prevention and Control of Noncommunicable Diseases 2013–2020, which aims for a 30% reduction in tobacco use by 2025.

To aid nations in achieving the tobacco use reduction goal, WHO has introduced the MPOWER measure and has constantly monitored progress in each country. The policy entails six main domains with varying benchmarks to identify if the measures are weak, minimal, moderate, or complete: M—monitor tobacco use and prevention policies; P—protect people from tobacco smoke; O—offer help to quit tobacco use; W—warn about the dangers of tobacco; E—enforce bans on tobacco advertising, promotion, and sponsorship; and R—raise taxes on tobacco.

Within the Western Pacific region, 24 countries have reached the highest level of achievement in at least one of these six measures. Furthermore, although no country in the region has achieved the highest level in all domains, most of them have attained at least the moderate recommendations, with 67% of countries in the region having recent and representative data for tobacco use; 56% with smoke-free legislation covering most public places; 78% have financed cessation services; 63% have appropriate health warnings; 89% have banned tobacco advertising and some or all forms of promotion and sponsorship; and 56% have taxes that comprise at least 51% of the retail price of cigarettes.

A 2018 progress review specific to the Western Pacific region found that more than 80% of the member countries had implemented campaigns to advocate for tobacco control and have provided brief cessation advice training to primary health-care workers. This update showed a two-fold improvement compared with 2014. Furthermore, 18 of 27 countries with sufficient data had high compliance with the bans on advertising, promotion, and sponsorship. Australia has also implemented annual tax increases of 12•5% to cigarette sales since 2013, which has proven effective in reducing smoking prevalence. In China, national mass media campaigns to warn people of the effects of smoking have also been used to deter smokers. Beyond national-level efforts, key stakeholders such as city councils, tourism operators, and tourist sites have also led the charge in many of these countries by initiating smoke-free regulations to dissuade smokers and protect their communities from second-hand smoke.

Despite the success of these strategies in reducing tobacco use, the current rate of decline in the Western Pacific region is insufficient to meet the 2025 WHO Global Action Plan target. Furthermore, new challenges and barriers affecting the efforts to reduce tobacco use are rapidly emerging. A serious problem is the increasing popularity of electronic nicotine delivery devices, such as e-cigarettes and vaping devices. The tobacco industry's efforts to brand and market these devices as less harmful than, and as cessation aids for, traditional cigarettes have contributed to their rising uptake.

In response to these challenges, the Western Pacific region has set a new target of a 30% reduction in tobacco use by 2030. Along with this target, part of the regional action plan focuses on preparing to combat the new challenges in tobacco control. To meet the new goal, countries of the region need to move beyond agreeing to the tobacco control measures and toward the strategies' active implementation and enforcement stages. While working on feasible changes, member nations need to aim for the highest degree of the recommendations (as set by WHO) to use. Additionally, a focus on the future will help countries face the emerging challenges with a proactive approach rather than reacting to a much larger problem in the future. Acting on tobacco control now will help save millions of lives in the region, and as WHO calls on smokers to Commit to Quit, we call on the Western Pacific nations to Commit to Action.

*The Lancet Regional Health – Western Pacific*

